# Cost-effectiveness analysis of cadonilimab plus bevacizumab and chemotherapy for persistent, recurrent, or metastatic cervical cancer

**DOI:** 10.3389/fimmu.2025.1594786

**Published:** 2025-06-25

**Authors:** Kaixuan Wang, Shixian Liu, Ruixue Wang, Lei Dou, Jie Gao

**Affiliations:** ^1^ Maternal and Child Health Development Research Center, Shandong Provincial Maternal and Child Health Care Hospital Affiliated to Qingdao University, Jinan, China; ^2^ Department of Social Medicine and Health Management, School of Public Health, Cheeloo College of Medicine, Shandong University, Jinan, China; ^3^ Office of Discipline Inspection, Shandong Second Provincial General Hospital, Jinan, China

**Keywords:** cost-effectiveness, cadonilimab, cervical cancer, bevacizumab, chemotherapy

## Abstract

**Objective:**

The aim of this study is to investigate the cost-effectiveness of cadonilimab plus bevacizumab and chemotherapy in the first-line treatment for patients with persistent, recurrent, or metastatic cervical cancer from a healthcare system perspective in China.

**Methods:**

A partitioned survival model was established to estimate the total costs, quality-adjusted life-years (QALYs), and incremental cost-effectiveness ratio (ICER) over a 10-year time horizon. Clinical data was sourced from the COMPASSION-16 trial; direct medical costs and utilities were obtained from a public drug bidding database and published literature. The robustness of the model was assessed via scenario, one-way and probabilistic sensitivity analyses.

**Results:**

Cadonilimab plus bevacizumab and chemotherapy yielded an additional cost of $31,654.02, with an additional QALY of 0.36, resulted in an ICER of $88,533.51/QALY compared with bevacizumab and chemotherapy. Utility values of progression-free survival (PFS), patient weight and price of cadonilimab were the most influential parameter on ICER. The probability of cadonilimab plus bevacizumab and chemotherapy being cost-effective was 0% at the WTP threshold of $38,042.49 per QALY. When the price of cadonilimab reduced by 72%, cadonilimab plus bevacizumab and chemotherapy would represent an economically viable treatment regime.

**Conclusion:**

Cadonilimab plus bevacizumab and chemotherapy may not be a cost-effective option as the first-line treatment in persistent, recurrent, or metastatic cervical cancer.

## Introduction

Despite the introduction of screening and vaccination programs, cervical cancer remains the fourth most common cancer in terms of both incidence and mortality among women, with an estimated 660,000 new cases and 350,000 deaths worldwide in 2022 (1). China is also a high-incidence region for cervical cancer, with a rate of 21.81 cases per 100,000 individuals (2). At the time of initial diagnosis, 4.3% of patients were found to be at stage III or IV (3). For patients with persistent, recurrent, or metastatic cervical cancer, the five-year overall survival (OS) rate is below 20% (4). Over the past ten years, the incorporation of bevacizumab into platinum-based chemotherapy has emerged as the primary first-line standard of care for patients with recurrent or metastatic cervical cancer based on GOG240 trial (5, 6). However, the evidence from the GOG240 trial revealed that the median OS for patients receiving bevacizumab plus chemotherapy was still less than 17 months (5). Therefore, it is urgent to explore new treatments in order to maximize patient outcomes.

Cadonilimab, a bispecific antibody blocking both PD-1 and CTLA-4 pathways, was approved in China on June 29, 2022 for the treatment of recurrent or metastatic cervical cancer which has progressed on or after platinum-based chemotherapy (7). The COMPASSION-16 trial indicated that the addition of cadonilimab to bevacizumab and platinum-based chemotherapy significantly improved progression free survival (PFS) [hazard ratio (HR): 0.62, 95% confidence interval (CI): 0.49-0.80] and OS [hazard ratio (HR): 0.64, 95% confidence interval (CI): 0.48-0.86] versus bevacizumab and platinum-based chemotherapy in patients with persistent, recurrent, or metastatic cervical cancer (8). White blood cell count decreased, anemia, and neutrophil count decreased were the most common treatment-related adverse events (AEs) (8).

Although the combination of cadonilimab, bevacizumab, and chemotherapy provided promising clinical benefits and manageable safety profile, the escalated cost associated with this particular therapeutic approach may hinder its widespread availability and impose a heavier financial burden on patients. Therefore, the study aimed to evaluate the cost-effectiveness of cadonilimab plus bevacizumab and platinum-based chemotherapy versus bevacizumab and platinum-based chemotherapy as the first-line treatment for persistent, recurrent, or metastatic cervical cancer from Chinese healthcare system perspective.

## Methods

This economic evaluation was conducted utilizing modelling techniques and published literature, and the approval of the institutional research ethics board was not required as no real human participants or animals were involved. This study adhered to consolidated health economic evaluation reporting standards 2022 (CHEERS 2022) (9) (**Supplementary Table 1**).

### Patients and intervention

The target patient population was aligned with the cohort enrolled in the COMPASSION-16 trial, an open-label, phase 3 trial conducted across 59 clinical sites in China (8). Eligible patients were women aged 18–75 years with persistent, recurrent, or metastatic (stage IVB) cervical cancer who have not received prior systemic therapy (8). The intervention group received cadonilimab (10 mg/kg) in combination with chemotherapy (cisplatin [50 mg/m^2^] or carboplatin [area under the curve 4-5] plus paclitaxel [175 mg/m^2^]), with or without bevacizumab (15 mg/kg). The control group received placebo plus chemotherapy with or without bevacizumab. The regimens were administered on day 1 of each 3-week cycle for six cycles. Subsequently, patients transitioned to maintenance therapy, consisting of cadonilimab or placebo, optionally in combination with bevacizumab, which was administered every 3 weeks thereafter (8). Treatment continued until disease progression, unacceptable toxicity or having received cadonilimab or placebo for 2 years (8). After disease progression, it was assumed that the remaining patients would receive best supportive care, given that subsequent treatment options had not been clearly defined in clinical trial (8).

### Model structure

**Figure 1:**
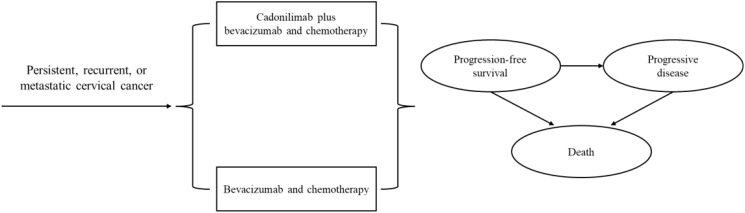
The structure of the partitioned survival model.

A partitioned survival model was developed to simulate disease progression using Excel 2019 and R 4.3.2 software. The model comprised three mutually exclusive health states: PFS, progressive disease (PD), and death (**Figure 1**). The cycle length was three weeks, aligning with the treatment protocol in clinical trial (8). The time horizon was established at 10 years to ensure that all cervical cancer patients entered the terminal state. Primary outcomes of the model included the total costs, quality-adjusted life-years (QALYs), and incremental cost-effectiveness ratio (ICER). The ICER was described as the incremental cost per additional QALY. To enhance the precision of the model results, a half-cycle correction was incorporated. According to China Guidelines for Pharmacoeconomic Evaluations, Costs and QALYs were discounted at an annual rate of 5% with a range of 0% to 8% (10). We used 3 times gross domestic product per capita ($38,042.49, in 2023) as the willingness-to-pay (WTP) threshold.

### Clinical data

The clinical efficacy and adverse reactions data were extracted from the COMPASSION-16 trial. GetData Graph Digitizer 2.26 was utilized to digitize Kaplan-Meier (KM) PFS and OS curves to reconstruct individual patient data (IPD), adhering to the algorithms outlined by Guyot et al. (11) (**Supplementary Table 2**). Seven parametric distributions, namely Exponential, Weibull, Gamma, Generalized Gamma, Gompertz, Log-normal and Log-logistic, were fitted to extrapolate the KM curves beyond the follow-up period of the clinical trial (12). The optimal distribution was determined through a comprehensive evaluation including visual assessments, Akaike information criterion (AIC) and Bayesian information criterion (BIC) (13). Based on this rigorous analysis, we selected Log-logistic distribution for PFS and OS curves in cadonilimab plus bevacizumab and chemotherapy group, while the Log-normal and Generalised Gamma distributions were chosen for PFS and OS curves in bevacizumab and chemotherapy group, respectively. More details concerning model fitting are presented in **Supplementary Tables 3, 4** and **Supplementary Figures 1-4**.

### Costs

Only direct medical expenses were considered, including medications, follow-up visit, laboratory test, imaging test, best supportive care, management of treatment-related severe AEs, and terminal care in end-of-life. Drug pricing information was sourced from the 2024 average bid-winning prices announced by YaoZH database (14). For dosage estimation, standardized patient parameters were assumed, including an average body weight of 60 kg, a body surface area (BSA) of 1.64 m² (15), and a creatinine clearance rate of 70 mL/min, which was specifically used for calculating the dose of carboplatin (15). Other associated costs were derived from published literature (15–18). Routine laboratory examination was conducted once per cycle. Abdominal computed tomography (CT) was performed every 6 weeks during the first 24 weeks, every 9 weeks from week 25 to week 51, and every 12 weeks thereafter until disease progression (8). Only grade 3–5 AEs with an incidence of greater than 5% were considered (8). All costs were standardized to 2024 US dollars using the 2023 average exchange rate of 1 USD = 7.0467 RMB.

### Utilities

Utility values were assigned to each health state, ranging from 0 (representing death) to 1 (representing perfect health). Because the COMPASSION-16 trial lacked data on quality of life, the utility values for the PFS and PD health states were derived from another published economic evaluation, in which the data were measured by the EuroQol five dimensions health status questionnaire (EQ-5D-3L) and US-specific value algorithm (19). In addition, disutility values caused by grade 3–5 AEs were obtained from relevant literature (18, 20, 21). All costs and utilities are shown in **Table 1**.

**Table 1:** Basic parameters input to the model and the ranges of the sensitivity analysis.

Parameters	Baseline value	Range		Distribution	Reference
		Minimum	Maximum		
Cost inputs (US $)					
Cadonilimab(1mg)	2.11	1.69	2.53	Gamma	(14)
Cisplatin(1mg)	0.20	0.16	0.24	Gamma	(14)
Carboplatin(1mg)	0.17	0.14	0.20	Gamma	(14)
Paclitaxel(1mg)	0.74	0.59	0.88	Gamma	(14)
Bevacizumab(1mg)	1.56	1.25	1.88	Gamma	(14)
Cost of best supportive care	337.66	270.13	405.19	Gamma	(15)
Cost of follow-up	55.63	44.50	66.75	Gamma	(15)
Cost of routine laboratory examinations	92.54	74.03	111.05	Gamma	(15)
Cost of abdominal CT	105.95	84.76	127.14	Gamma	(15)
Cost of terminal care in end-of-life	1460.30	1055.30	2085.70	Gamma	(16)
Cost of white blood cell count decreased	456.30	365.04	547.56	Gamma	(17)
Cost of anemia	497.62	398.10	597.14	Gamma	(17)
Cost of neutrophil count decreased	523.27	418.62	627.93	Gamma	(17)
Cost of platelet count decreased	1375.32	1100.26	1650.39	Gamma	(18)
Cost of hypokalemia	2908.92	2327.14	3490.70	Gamma	(18)
Cost of hypertriglyceridemia	102.47	81.97	122.96	Gamma	Assumption
Cost of lymphocyte count decreased	105.03	84.02	126.04	Gamma	(18)
Cost of hypertension	102.47	81.97	122.96	Gamma	(18)
Utility inputs					
PFS	0.817	0.654	0.980	Beta	(19)
PD	0.779	0.623	0.935	Beta	(19)
Disutility inputs					
White blood cell count decreased	0.200	0.160	0.240	Beta	(18)
Anemia	0.120	0.096	0.144	Beta	(20)
Neutrophil count decreased	0.150	0.120	0.180	Beta	(21)
Platelet count decreased	0.110	0.088	0.132	Beta	(18)
Hypokalemia	0.030	0.024	0.036	Beta	(18)
Hypertriglyceridemia	0.080	0.064	0.096	Beta	Assumption
Lymphocyte count decreased	0.200	0.160	0.240	Beta	(18)
Hypertension	0.080	0.064	0.096	Beta	(18)
Risk of severe adverse events in CBC group					
White blood cell count decreased	28.30%	22.64%	33.96%	Beta	(8)
Anemia	15.90%	12.72%	19.08%	Beta	(8)
Neutrophil count decreased	40.70%	32.56%	48.84%	Beta	(8)
Platelet count decreased	14.20%	11.36%	17.04%	Beta	(8)
Hypokalemia	5.80%	4.64%	6.96%	Beta	(8)
Hypertriglyceridemia	6.20%	4.96%	7.44%	Beta	(8)
Lymphocyte count decreased	7.10%	5.68%	8.52%	Beta	(8)
Hypertension	6.60%	5.28%	7.92%	Beta	(8)
Risk of severe adverse events in BC group					
White blood cell count decreased	36.10%	28.88%	43.32%	Beta	(8)
Anemia	24.20%	19.36%	29.04%	Beta	(8)
Neutrophil count decreased	46.10%	36.88%	55.32%	Beta	(8)
Platelet count decreased	12.30%	9.84%	14.76%	Beta	(8)
Lymphocyte count decreased	6.80%	5.44%	8.16%	Beta	(8)
Hypertension	9.60%	7.68%	11.52%	Beta	(8)
proportion of platinum therapy use during the trial					
CBC group	58.56%	46.85%	70.27%	Beta	(8)
BC group	55.16%	44.13%	66.19%	Beta	(8)
proportion of bevacizumab use during the trial					
CBC group	59.91%	47.93%	71.89%	Beta	(8)
BC group	59.19%	47.35%	71.03%	Beta	(8)
Others					
Patient weight (kg)	60.00	48.00	72.00	Gamma	(15)
Body surface area(m^2^)	1.64	1.31	1.97	Gamma	(15)
Creatinine clearance	70.00	56.00	84.00	Gamma	(15)
Discount rate	0.05	0.00	0.08	Beta	(10)

CBC, cadonilimab plus bevacizumab and chemotherapy; BC, bevacizumab and chemotherapy; PFS, progression-free survival; PD, progressive disease.

### Scenario analyses

Firstly, a price reduction strategy was employed to determine the most reasonable pricing for cadonilimab. Secondly, we adjusted a time horizon of 5 years to examine its impact on the ICER.

### Sensitivity analyses

To assess the robustness of the base-case results, both one-way sensitivity analysis (OWSA) and probabilistic sensitivity analysis (PSA) were conducted. In the OWSA, the impact of various input parameters on the ICER was evaluated by varying them within a range of ±20% or 95% confidence intervals of the base case value (22). The results of OWSA were visually presented using Tornado diagrams. For the PSA, a Monte Carlo simulation was performed with 10,000 iterations, where key parameters were simultaneously sampled from predefined distributions (23). Specifically, Gamma distributions were used for costs, while beta distributions were chosen for incidence, utilities, and disutilities (24). The results of the PSA were presented in the form of an incremental cost-effectiveness scatter plot and a cost-effectiveness acceptability curve.

## Results

### Base case results

Compared with bevacizumab and chemotherapy group, cadonilimab plus bevacizumab and chemotherapy group provided incremental cost of $31,654.02, with additional QALY of 0.36, resulting in an ICER of $88,533.51 per QALY in the treatment for persistent, recurrent, or metastatic cervical cancer (**Table 2**).

**Table 2:** Base case results.

Strategy	Total costs, $	Total QALYs	Incremental costs, $	Incremental QALYs	ICER
BC group	44,966.18	2.22	–	–	–
CBC group	76,559.13	2.58	31,592.95	0.36	88,362.70

CBC, cadonilimab plus bevacizumab and chemotherapy; BC, bevacizumab and chemotherapy; QALYs, quality-adjusted life years; ICER, incremental cost-effectiveness ratio.

### Scenario analyses

**Figure 2:**
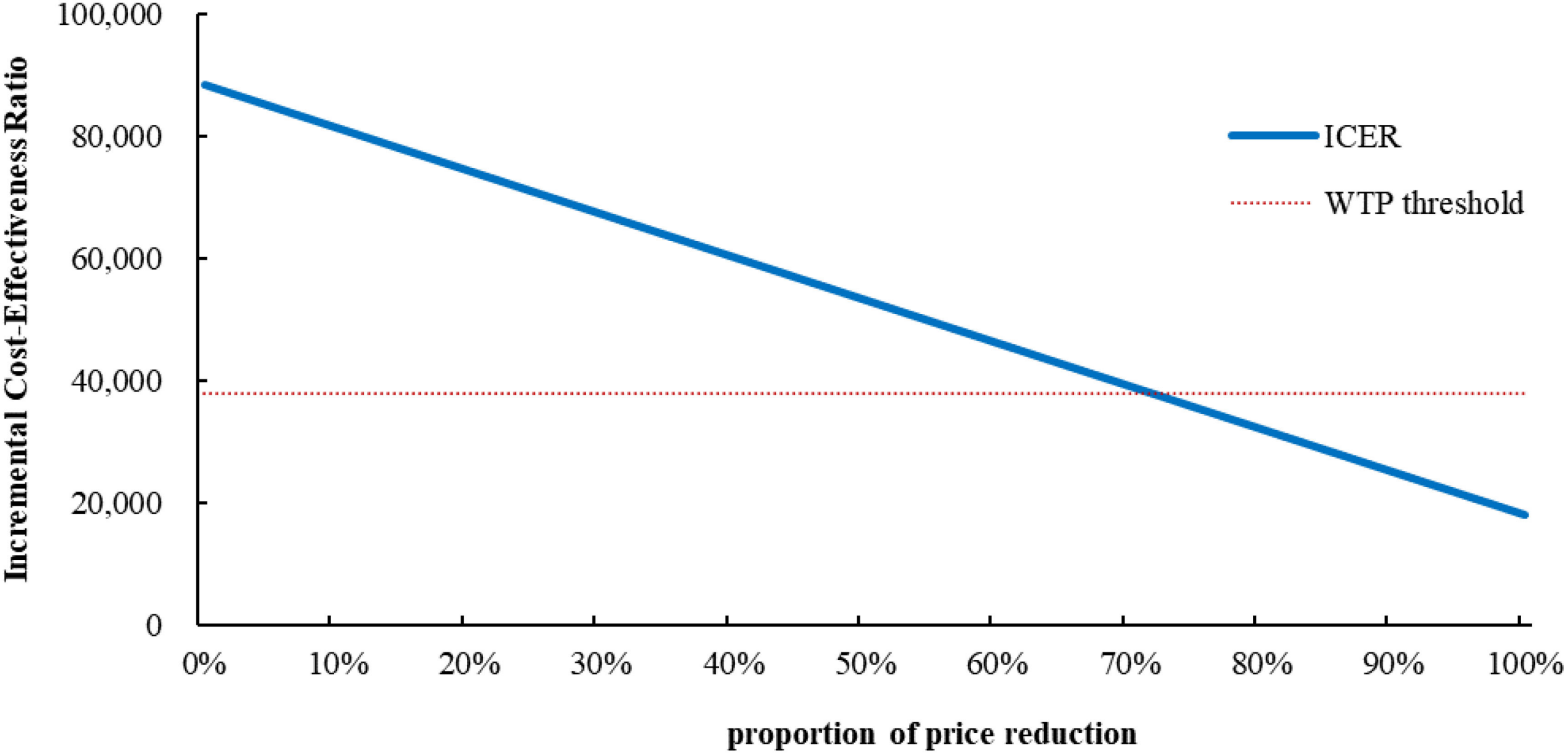
Scenario analysis results for price reductions of cadonilimab. ICER, incremental cost-effectiveness ratio; WTP, willingness to Pay.

Only if the price of cadonilimab was reduced by 72% to $0.59 per mg or more, the cadonilimab plus bevacizumab and chemotherapy group would be cost-effective (**Figure 2**). At the 5-year time horizon, the ICER of cadonilimab plus bevacizumab and chemotherapy group were $101,638.04 per QALYs (**Supplementary Table 5**).

### Sensitivity analyses

**Figure 3:**
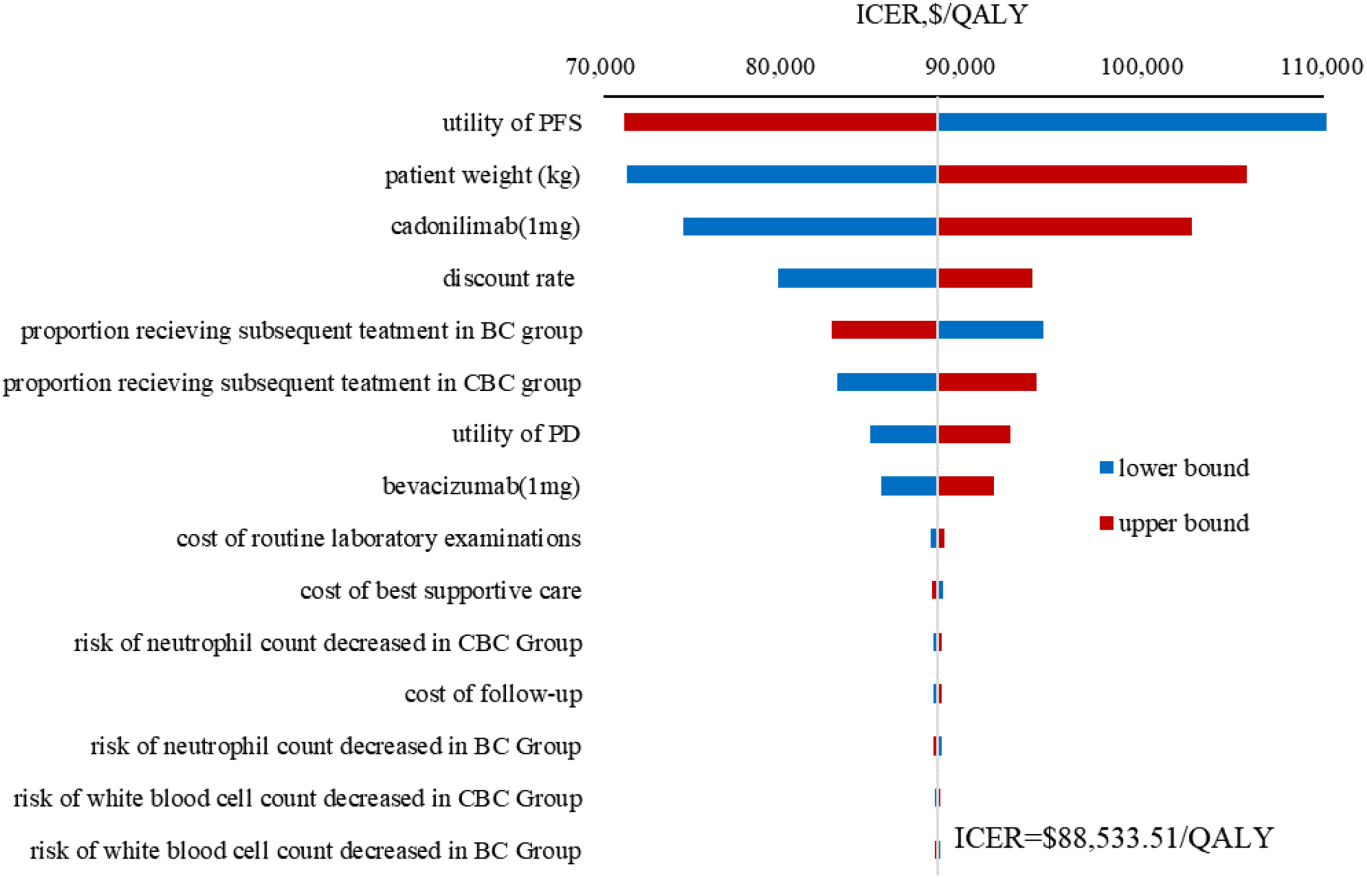
Tornado diagram of one-way sensitivity analysis results. ICER, incremental cost-effectiveness ratio; QALY, quality-adjusted life year; CBC, cadonilimab plus bevacizumab and chemotherapy; BC, bevacizumab and chemotherapy; PFS, progression-free survival; PD, progressive disease.

**Figure 4:**
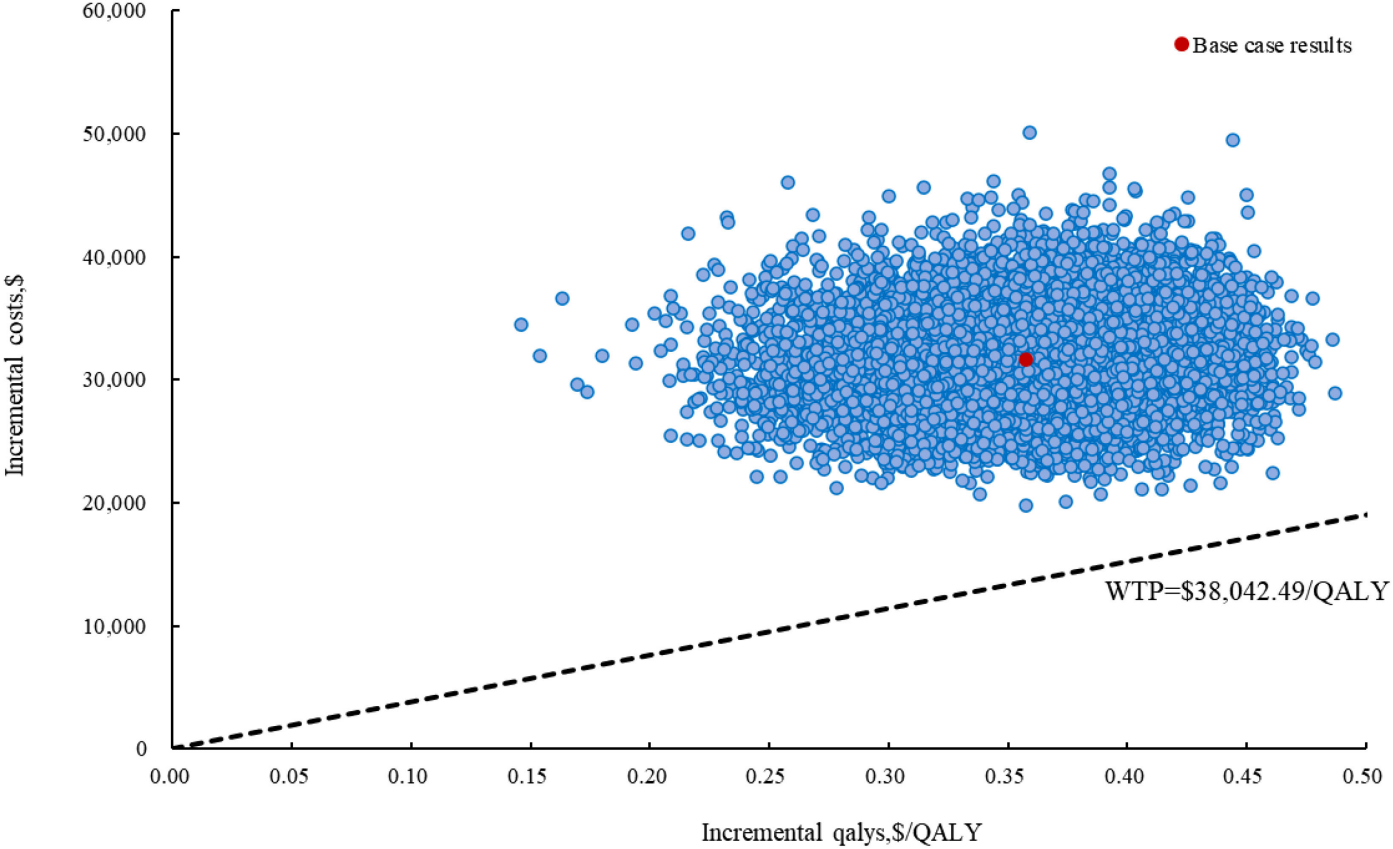
The incremental cost-effectiveness scatter plot of cadonilimab plus bevacizumab and chemotherapy compared to bevacizumab and chemotherapy in China. QALY, quality-adjusted life year; WTP, willingness to Pay.

**Figure 5:**
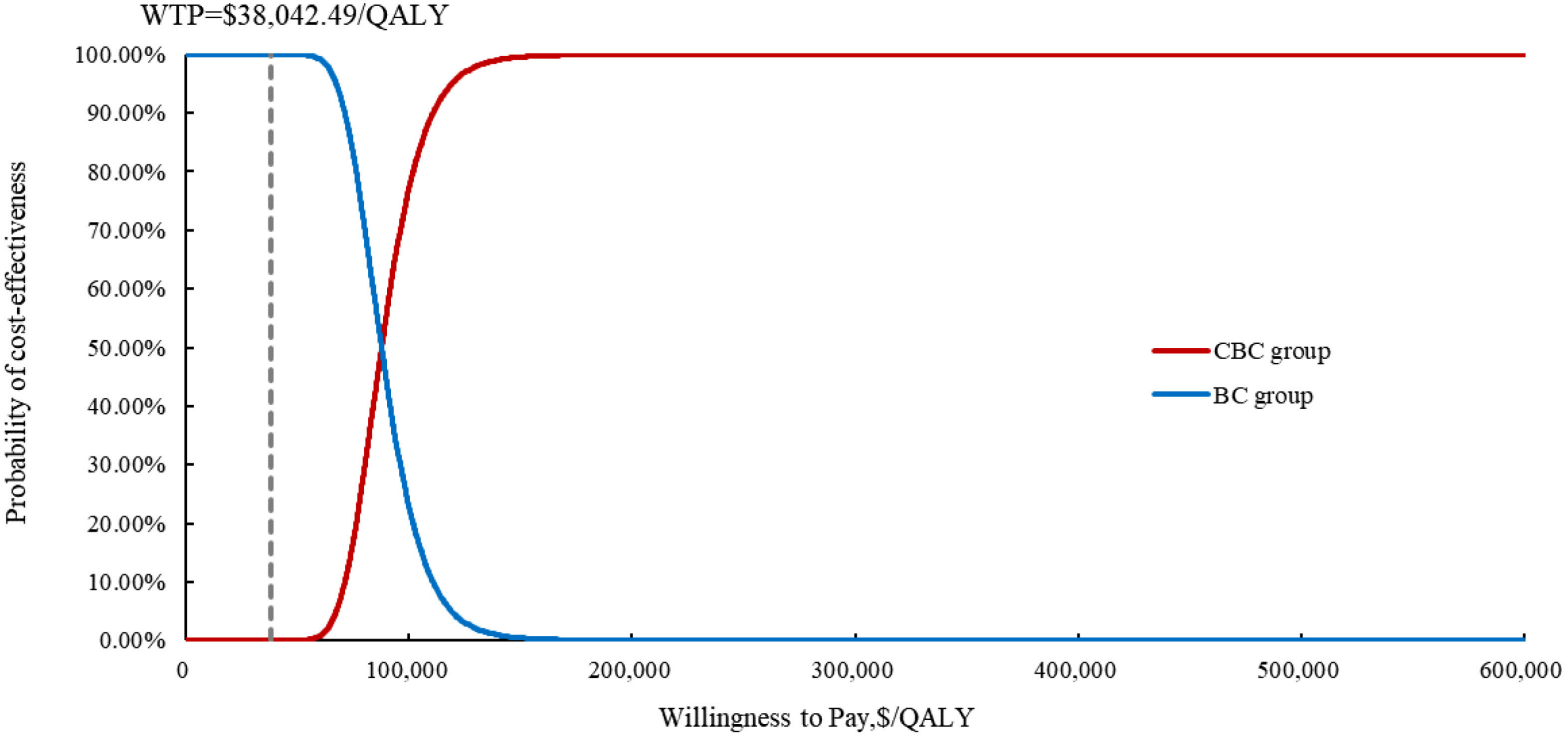
The cost-effectiveness acceptability curve of the probabilistic sensitivity analysis results. CBC, cadonilimab plus bevacizumab and chemotherapy; BC, bevacizumab and chemotherapy; QALY, quality-adjusted life year; WTP, willingness to Pay.

The results of the OWSA indicated that utility of PFS, patient weight, the price of cadonilimab, discount rate were the most influential variables on the ICER (**Figure 3**). However, the ICER is always higher than WTP threshold no matter how these variables were changed within a given range, indicating that our model was robust. The results of the PSA indicated that the probability of cadonilimab being cost-effective was 0% at the WTP threshold of 3 times GDP per capita ($38,042.49) (**Figures 4**, **5**).

## Discussion

Cadonilimab has been approved by National Medical Products Administration for the treatment of cervical cancer and advanced gastric or gastroesophageal junction (G/GEJ) adenocarcinoma (7, 8, 25). To our knowledge, this study represents the first model analysis to evaluate the cost-effectiveness of cadonilimab plus bevacizumab and platinum-based chemotherapy in the first-line treatment of persistent, recurrent, or metastatic cervical cancer by incorporating the most recent clinical evidence from the perspective of Chinese healthcare system. The ICER of cadonilimab plus bevacizumab and chemotherapy was calculated to be $88,533.51 per QALY compared with bevacizumab and chemotherapy, which exceeded doubling the China’s WTP threshold.

Utility of PFS, patient weight, the unit price of cadonilimab, and discount rate were the most influential parameters within the model, but variations in these parameters did not alter the conclusion, thereby demonstrating the robustness of our model. Due to the absence of quality of life data in COMPASSION-16 trial, the utility values were derived from a cost-effectiveness analysis (26) evaluating pembrolizumab for unresectable or metastatic endometrial cancer referred to Lei et al. (19) and Shi et al. (27). Further cost-effectiveness analysis should be conducted based on the health utility values of different treatments. Our results were consistent with previously published studies (28, 29) in identifying patient weight as a key variable given that cadonilimab was administered based on body weight (8). Consequently, for overweight or obese patients, the use of cadonilimab was disadvantageous as the increased dosage and associated costs required. Despite the excellent clinical efficacy associated with cadonilimab-based combination therapy, it did not fulfil the cost-effectiveness criteria due to increased utilization of healthcare resources. An adjustment in the price of this drug could have a substantial influence on the ICER. Scenario analysis results indicated that when the price of cadonilimab reduced to $0.59 per mg (equivalent to 28% of the current price), the ICER for cadonilimab group was $37,803.74 per QALY lower than the WTP threshold of $38,042.49.

In addition to cadonilimab, atezolizumab and pembrolizumab were other available immune checkpoint inhibitors (ICIs) as the first-line treatment for persistent, recurrent, or metastatic cervical cancer (30, 31). Cai et al. compared the cost-effectiveness of atezolizumab plus bevacizumab and chemotherapy versus bevacizumab and chemotherapy based on the BEATcc clinical trial from the Chinese healthcare system perspective, and showed that atezolizumab in combination with bevacizumab and chemotherapy was unlikely to be a cost-effective option (15). Three other analyses (19, 32, 33) also demonstrated that the treatment regime was not cost-effective for patients in the US. Two studies evaluated the cost-effectiveness of pembrolizumab plus bevacizumab and chemotherapy using efficacy data from the KEYNOTE-826 clinical trial in China and concluded that this therapeutic regime may not be a cost-effective primary strategy with an ICER of $52,765.69 per QALY (34) and $64,338.19 QALY (35), respectively, which in consistent with findings from another two analysis in the US (27, 36). However, Monk et al. suggested that the regime was proved to be cost-effective in the US, which might be attributed to variations in cost measurements and model constructions (37).

Apart from cervical cancers, G/GEJ adenocarcinoma was also important indications for cadonilimab approved by National Medical Products Administration based on a randomized controlled phase 3 study (25). In the COMPASSION-16 trial, 610 patients from 75 centers in China were randomized to receive cadonilimab or placebo plus chemotherapy (25). The results showed that cadonilimab plus chemotherapy significantly prolonged median PFS [hazard ratio (HR): 0.55, 95% confidence interval (CI): 0.44-0.65] and OS (14.1 versus 11.1 months; HR: 0.66, 95% CI: 0.54-0.81) compared with chemotherapy alone as the first-line treatment in advanced G/GEJ adenocarcinoma (25). However, to date, no cost-effectiveness analysis has been conducted to explore whether the survival benefit from cadonilimab could be matched by its pricing. Therefore, future studies could focus on the cost-effectiveness of cadonilimab for advanced G/GEJ adenocarcinoma, which was crucial for the clinical oncologists and healthcare policy makers.

Our model has several limitations. First, long-term efficacy data of the COMPASSION-16 trial was extrapolated using parametric survival models. Further validation using the update follow-up data is necessary because of the inherent uncertainty associated with the methodology. Second, since quality of life data was not reported in the COMPASSION-16 trial, we derived utility values from published literature. But the sensitivity analysis indicated that alterations in each utility values had not substantially altered the results. Third, the management costs and disutility values associated with grade 1 or 2 treatment-related AEs were not included in the model, considering their minimal impact on base-case results. Fourth, as subsequent treatments were not accounted for in the clinical trials, we assumed that BSC was the primary subsequent regimen, which may be distinct from realistic clinical choices.

## Conclusion

The combination of cadonilimab with bevacizumab and chemotherapy might not be cost-effectiveness compared with bevacizumab and chemotherapy in the first-line treatment for persistent, recurrent, or metastatic cervical cancer in China. Nevertheless, it is suggested that via reducing the price of cadonilimab to 28% of the current price, the regime could be a cost-effective option at the current WTP threshold.

## Data Availability

The original contributions presented in the study are included in the article/**Supplementary Material**. Further inquiries can be directed to the corresponding author.
