# Supplementary material for: Cost-effectiveness analysis of cadonilimab plus bevacizumab and chemotherapy for persistent, recurrent, or metastatic cervical cancer
Source: Front Immunol. 2025 Jun 25;16:1594786. doi: 10.3389/fimmu.2025.1594786 (PMC12237631; doi:10.3389/fimmu.2025.1594786)
Supplement: Supplementary file 1 [file Table1.doc]

**Cost-effectiveness Analysis of Cadonilimab plus Bevacizumab and Chemotherapy for Persistent, Recurrent, or Metastatic Cervical Cancer**

Kaixuan Wang^1^, Shixian Liu^2^, Ruixue Wang^3^, Lei Dou^2^, Jie Gao^1*^

^1^Shandong Provincial Maternal and Child Health Care Hospital Affiliated to Qingdao University, Jinan, China

^2^Department of Social Medicine and Health Management, School of Public Health, Cheeloo College of Medicine, Shandong University, Jinan, China

^3^Shandong Second Provincial General Hospital, Jinan, China

^*^Correspondence: Jie Gao

E-mail addresses: agao1224@163.com

**Supplementary Materials**

**Supplementary Table 1.** CHEERS Checklist 2022

**Supplementary Table 2.** Individual patient data for cadonilimab plus bevacizumab and chemotherapy versus bevacizumab and chemotherapy

**Supplementary Table 3.** Summary of statistical goodness-of-fit of Kaplan-Meier curves

**Supplementary Table 4.** Long-term PFS and OS data

**Supplementary Table 5**. Scenario analysis results

**Supplementary Figure 1.** The reconstructed Kaplan-Meier PFS curves of cadonilimab plus bevacizumab and chemotherapy

**Supplementary Figure 2.** The reconstructed Kaplan-Meier OS curves of cadonilimab plus bevacizumab and chemotherapy

**Supplementary Figure 3.** The reconstructed Kaplan-Meier PFS curves of bevacizumab and chemotherapy

**Supplementary Figure 4.** The reconstructed Kaplan-Meier OS curves of bevacizumab and chemotherapy

**Supplementary Table 1.** CHEERS Checklist 2022

| Section | Item  No | Guidance for reporting | Reported |
| --- | --- | --- | --- |
| **Title** | | | |
| Title | 1 | Identify the study as an economic evaluation and specify the interventions being compared | Yes |
| **Abstract** | | | |
| Abstract | 2 | Provide a structured summary that highlights context, key  methods, results, and alternative analyses | Yes |
| **Introduction** | | | |
| Background and objectives | 3 | Give the context for the study, the study question, and its practical relevance for decision making in policy or practice | Yes |
| **Methods** | | | |
| Health economic analysis plan | 4 | Indicate whether a health economic analysis plan was developed and where available | Yes |
| Study population | 5 | Describe characteristics of the study population (such as age range, demographics, socioeconomic, or clinical characteristics) | Yes |
| Setting and location | 6 | Provide relevant contextual information that may influence findings | Yes |
| Comparators | 7 | Describe the interventions or strategies being compared and why chosen | Yes |
| Perspective | 8 | State the perspective(s) adopted by the study and why chosen | Yes |
| Time horizon | 9 | State the time horizon for the study and why appropriate | Yes |
| Discount rate | 10 | Report the discount rate(s) and reason chosen | Yes |
| Selection of outcomes | 11 | Describe what outcomes were used as the measure(s) of benefit(s) and harm(s) | Yes |
| Measurement of outcomes | 12 | Describe how outcomes used to capture benefit(s) and harm(s) were measured | Yes |
| Valuation of outcomes | 13 | Describe the population and methods used to measure and value outcomes | Yes |
| Measurement and valuation of resources and costs | 14 | Describe how costs were valued | Yes |
| Currency, price date, and conversion | 15 | Report the dates of the estimated resource quantities and unit costs, plus the currency and year of conversion | Yes |
| Rationale and description of model | 16 | If modelling is used, describe in detail and why used. Report if the model is publicly available and where it can be accessed | Yes |
| Analytics and assumptions | 17 | Describe any methods for analysing or statistically transforming data, any extrapolation methods, and approaches for validating  any model used | Yes |
| Characterising heterogeneity | 18 | Describe any methods used for estimating how the results of the study vary for subgroups | Yes |
| Characterising distributional effects | 19 | Describe how impacts are distributed across different individuals or adjustments made to reflect priority populations | Yes |
| Characterising uncertainty | 20 | Describe methods to characterise any sources of uncertainty in the analysis | Yes |
| Approach to engagement with patients and others affected by the study | 21 | Describe any approaches to engage patients or service recipients, the general public, communities, or stakeholders (such as clinicians or payers) in the design of the study | Yes |
| **Results** | | | |
| Study parameters | 22 | Report all analytic inputs (such as values, ranges, references) including uncertainty or distributional assumptions | Yes |
| Summary of main results | 23 | Report the mean values for the main categories of costs and outcomes of interest and summarise them in the most appropriate overall measure | Yes |
| Effect of uncertainty | 24 | Describe how uncertainty about analytic judgments, inputs, or projections affect findings. Report the effect of choice of discount rate and time horizon, if applicable | Yes |
| Effect of engagement with patients  and others affected by the study | 25 | Report on any difference patient/service recipient, general  public, community, or stakeholder involvement made to the approach or findings of the study | Yes |
| **Discussion** | | | |
| Study findings, limitations, generalisability, and current knowledge | 26 | Report key findings, limitations, ethical or equity considerations not captured, and how these could affect patients, policy, or practice | Yes |
| Other relevant information Source of funding | 27 | Describe how the study was funded and any role of the funder in the identification, design, conduct, and reporting of the analysis | Yes |
| Conflicts of interest | 28 | Report authors conflicts of interest according to journal or  International Committee of Medical Journal Editors requirements | Yes |

**Supplementary Table 2.** Individual patient data for cadonilimab plus bevacizumab and chemotherapy versus bevacizumab and chemotherapy

| No | PFS | | | | OS | | | |
| --- | --- | --- | --- | --- | --- | --- | --- | --- |
|  | CBC | | BC | | CBC | | BC | |
|  | Time | Rate | Time | Rate | Time | Rate | Time | Rate |
| 1 | 0.00 | 100.00 | 0.00 | 100.00 | 0.00 | 100.00 | 0.00 | 100.00 |
| 2 | 0.56 | 100.00 | 1.04 | 100.00 | 0.58 | 100.00 | 1.77 | 100.00 |
| 3 | 0.66 | 99.55 | 1.18 | 99.55 | 0.67 | 99.53 | 2.14 | 99.53 |
| 4 | 0.79 | 99.09 | 1.99 | 95.76 | 0.80 | 99.19 | 2.29 | 98.95 |
| 5 | 1.52 | 98.64 | 2.16 | 95.61 | 2.94 | 98.72 | 3.54 | 98.72 |
| 6 | 1.91 | 97.58 | 2.49 | 95.31 | 3.93 | 98.25 | 3.80 | 98.25 |
| 7 | 2.76 | 97.13 | 2.99 | 92.59 | 4.06 | 97.79 | 4.50 | 97.32 |
| 8 | 2.89 | 96.67 | 3.09 | 91.23 | 4.13 | 97.32 | 4.54 | 96.86 |
| 9 | 2.93 | 96.22 | 3.38 | 91.07 | 4.32 | 96.86 | 4.56 | 96.74 |
| 10 | 3.05 | 95.76 | 3.55 | 90.17 | 4.97 | 96.39 | 5.19 | 95.93 |
| 11 | 3.45 | 95.16 | 3.82 | 89.71 | 6.03 | 95.46 | 5.36 | 95.46 |
| 12 | 3.90 | 94.71 | 4.15 | 89.26 | 6.42 | 94.99 | 5.68 | 94.99 |
| 13 | 3.94 | 94.25 | 4.24 | 88.35 | 6.59 | 94.53 | 6.05 | 94.53 |
| 14 | 4.05 | 93.80 | 4.28 | 88.20 | 7.54 | 93.60 | 6.20 | 93.48 |
| 15 | 4.11 | 93.34 | 4.34 | 86.38 | 7.93 | 93.13 | 6.46 | 92.78 |
| 16 | 4.17 | 91.83 | 4.53 | 85.93 | 8.56 | 92.67 | 6.53 | 92.20 |
| 17 | 4.28 | 90.77 | 4.73 | 84.87 | 8.64 | 92.20 | 6.59 | 92.08 |
| 18 | 4.30 | 90.32 | 4.84 | 84.12 | 8.93 | 91.73 | 6.72 | 91.27 |
| 19 | 4.34 | 89.86 | 5.21 | 83.96 | 9.18 | 91.27 | 6.90 | 90.57 |
| 20 | 4.53 | 89.41 | 5.48 | 80.03 | 9.40 | 90.80 | 6.96 | 90.34 |
| 21 | 4.63 | 88.96 | 5.52 | 78.06 | 9.47 | 90.34 | 7.13 | 89.41 |
| 22 | 4.96 | 88.35 | 5.58 | 76.25 | 10.03 | 89.87 | 8.17 | 85.56 |
| 23 | 5.40 | 86.99 | 5.63 | 76.10 | 10.14 | 89.41 | 8.30 | 84.63 |
| 24 | 5.52 | 85.48 | 5.69 | 73.98 | 10.39 | 88.36 | 8.50 | 83.59 |
| 25 | 5.54 | 84.12 | 5.79 | 71.10 | 10.44 | 87.89 | 8.78 | 82.65 |
| 26 | 5.58 | 83.51 | 6.46 | 68.53 | 10.46 | 87.43 | 9.10 | 82.19 |
| 27 | 5.63 | 83.06 | 6.58 | 67.93 | 10.59 | 86.96 | 9.49 | 81.72 |
| 28 | 5.73 | 82.00 | 6.62 | 67.62 | 10.70 | 86.50 | 10.20 | 79.63 |
| 29 | 5.75 | 81.54 | 6.85 | 67.02 | 10.83 | 86.03 | 10.29 | 79.28 |
| 30 | 5.79 | 80.48 | 6.91 | 65.96 | 11.22 | 85.56 | 10.44 | 78.81 |
| 31 | 5.98 | 79.58 | 6.98 | 65.66 | 11.41 | 85.10 | 10.49 | 78.23 |
| 32 | 6.58 | 78.97 | 7.10 | 65.05 | 11.54 | 84.63 | 10.68 | 77.30 |
| 33 | 7.00 | 78.52 | 7.39 | 62.93 | 11.97 | 84.05 | 10.81 | 77.07 |
| 34 | 7.16 | 78.06 | 7.45 | 62.63 | 11.99 | 83.59 | 11.29 | 75.79 |
| 35 | 7.35 | 77.61 | 7.54 | 61.57 | 12.02 | 83.24 | 11.83 | 74.27 |
| 36 | 7.43 | 76.55 | 7.60 | 60.06 | 12.04 | 83.12 | 12.28 | 72.64 |
| 37 | 7.54 | 76.10 | 7.62 | 59.61 | 12.73 | 82.65 | 12.54 | 71.59 |
| 38 | 7.56 | 75.04 | 7.66 | 58.09 | 12.79 | 82.19 | 12.80 | 70.66 |
| 39 | 7.62 | 74.58 | 7.79 | 56.58 | 12.84 | 81.72 | 13.23 | 69.27 |
| 40 | 7.68 | 73.83 | 7.83 | 54.92 | 13.03 | 81.26 | 13.30 | 69.03 |
| 41 | 7.79 | 72.47 | 7.91 | 52.95 | 13.16 | 80.79 | 13.54 | 68.57 |
| 42 | 7.83 | 70.95 | 8.01 | 51.44 | 13.23 | 80.33 | 14.18 | 67.87 |
| 43 | 7.89 | 69.89 | 8.30 | 49.47 | 13.36 | 79.86 | 14.23 | 67.52 |
| 44 | 8.08 | 68.84 | 8.51 | 48.41 | 13.44 | 79.39 | 14.81 | 66.36 |
| 45 | 8.12 | 67.93 | 8.78 | 47.96 | 13.49 | 78.81 | 15.07 | 65.89 |
| 46 | 8.70 | 66.87 | 9.03 | 47.96 | 13.64 | 78.35 | 15.11 | 65.31 |
| 47 | 9.40 | 66.26 | 9.47 | 46.90 | 13.79 | 77.88 | 15.18 | 64.26 |
| 48 | 9.47 | 65.81 | 9.51 | 44.93 | 14.54 | 77.42 | 15.22 | 63.80 |
| 49 | 9.49 | 65.36 | 9.53 | 44.78 | 15.08 | 76.95 | 15.42 | 63.33 |
| 50 | 9.55 | 64.75 | 9.70 | 42.81 | 15.19 | 76.48 | 15.48 | 63.21 |
| 51 | 9.63 | 64.30 | 9.82 | 41.75 | 15.62 | 76.02 | 15.74 | 62.17 |
| 52 | 9.70 | 63.69 | 9.97 | 41.45 | 15.88 | 75.55 | 16.45 | 61.58 |
| 53 | 9.72 | 63.24 | 10.21 | 40.70 | 15.97 | 74.97 | 16.65 | 61.12 |
| 54 | 9.82 | 62.18 | 10.44 | 40.09 | 16.01 | 74.51 | 16.91 | 60.19 |
| 55 | 9.92 | 61.57 | 10.67 | 39.64 | 16.08 | 74.04 | 17.08 | 59.02 |
| 56 | 10.11 | 61.12 | 11.21 | 39.03 | 16.51 | 73.57 | 17.64 | 57.39 |
| 57 | 10.34 | 60.67 | 11.42 | 38.43 | 16.53 | 73.11 | 18.55 | 55.76 |
| 58 | 10.38 | 60.06 | 11.54 | 37.82 | 16.86 | 72.06 | 20.19 | 55.18 |
| 59 | 10.42 | 59.61 | 11.73 | 36.61 | 17.55 | 71.59 | 21.56 | 53.55 |
| 60 | 10.67 | 59.00 | 13.37 | 34.95 | 17.87 | 71.13 | 22.38 | 51.92 |
| 61 | 10.82 | 58.55 | 13.52 | 34.34 | 18.67 | 70.66 | 22.77 | 51.34 |
| 62 | 11.40 | 57.94 | 14.49 | 33.74 | 18.91 | 70.20 | 23.72 | 50.06 |
| 63 | 11.61 | 57.49 | 14.76 | 32.38 | 19.00 | 69.73 | 24.43 | 48.43 |
| 64 | 11.63 | 56.88 | 14.89 | 30.71 | 19.58 | 68.68 | 25.69 | 45.75 |
| 65 | 11.71 | 56.43 | 15.09 | 29.65 | 19.73 | 68.22 | 26.38 | 44.70 |
| 66 | 11.77 | 55.37 | 15.26 | 28.74 | 19.86 | 67.75 | 26.44 | 44.24 |
| 67 | 11.83 | 54.77 | 15.40 | 28.29 | 20.88 | 67.29 | 29.04 | 43.54 |
| 68 | 11.92 | 54.31 | 16.19 | 26.78 | 21.52 | 66.71 | 30.05 | 39.12 |
| 69 | 11.96 | 53.71 | 16.36 | 25.72 | 22.06 | 66.24 |  |  |
| 70 | 12.00 | 52.19 | 16.51 | 25.11 | 22.97 | 65.77 |  |  |
| 71 | 12.10 | 51.13 | 17.09 | 24.81 | 23.30 | 64.14 |  |  |
| 72 | 12.73 | 50.53 | 17.58 | 23.75 | 23.60 | 63.56 |  |  |
| 73 | 12.79 | 49.92 | 23.07 | 22.09 | 23.99 | 62.98 |  |  |
| 74 | 13.27 | 49.32 |  |  | 24.66 | 62.28 |  |  |
| 75 | 13.35 | 48.87 |  |  | 24.70 | 61.58 |  |  |
| 76 | 13.52 | 48.26 |  |  | 26.45 | 60.77 |  |  |
| 77 | 13.54 | 47.81 |  |  | 26.58 | 59.72 |  |  |
| 78 | 14.49 | 47.20 |  |  | 27.01 | 58.56 |  |  |
| 79 | 14.62 | 46.60 |  |  | 27.79 | 57.51 |  |  |
| 80 | 15.18 | 45.23 |  |  | 28.16 | 56.00 |  |  |
| 81 | 15.38 | 44.48 |  |  | 28.79 | 54.25 |  |  |
| 82 | 16.07 | 43.57 |  |  | 31.57 | 51.57 |  |  |
| 83 | 17.09 | 42.81 |  |  |  |  |  |  |
| 84 | 17.31 | 41.91 |  |  |  |  |  |  |
| 85 | 17.38 | 41.00 |  |  |  |  |  |  |
| 86 | 18.44 | 39.64 |  |  |  |  |  |  |
| 87 | 19.72 | 37.82 |  |  |  |  |  |  |
| 88 | 20.01 | 35.85 |  |  |  |  |  |  |
| 89 | 22.92 | 30.86 |  |  |  |  |  |  |
| CBC, cadonilimab plus bevacizumab and chemotherapy; BC, bevacizumab and chemotherapy; PFS, progression-free survival; OS, overall survival. | | | | | | | | |

**Supplementary Table 3.** Summary of statistical goodness-of-fit of Kaplan-Meier curves

|  | **Exponential** | **Weibull** | **Gamma** | **Generalised gamma** | **Gompertz** | **Lognormal** | **Log-logistic** |
| --- | --- | --- | --- | --- | --- | --- | --- |
| **Cadonilimab plus bevacizumab and chemotherapy-PFS** | | | | | | | |
| parameter 1 | 0.04669 | 1.536 | 1.9516 | 2.669 | 0.0584 | 2.6328 | 1.9150 |
| parameter 2 |  | 18.003 | 0.1165 | 0.892 | 0.0302 | 0.9242 | 13.6230 |
| parameter 3 |  |  |  | 0.124 |  |  |  |
| AIC | 887.9906 | 866.9890 | 863.2131 | 861.4396 | 880.3349 | 859.6012 | 859.5691* |
| BIC | 891.3933 | 873.7944 | 870.0184 | 871.6476 | 887.1402 | 866.4065 | 866.3744* |
| **Cadonilimab plus bevacizumab and chemotherapy-OS** | | | | | | | |
| parameter 1 | 0.01845 | 1.49 | 1.71633 | 3.657 | 0.04028 | 3.5669 | 1.6910 |
| parameter 2 |  | 40.769 | 0.04331 | 0.862 | 0.01101 | 1.1192 | 32.9010 |
| parameter 3 |  |  |  | 0.55 |  |  |  |
| AIC | 820.7957 | 809.3839 | 808.5545 | 810.2499 | 814.9386 | 810.4879 | 807.7857* |
| BIC | 824.1984 | 816.1892 | 815.3599 | 820.4579 | 821.7439 | 817.2932 | 814.5911* |
| **Bevacizumab and chemotherapy-PFS** | | | | | | | |
| parameter 1 | 0.07055 | 1.641 | 2.3461 | 2.145 | 0.07582 | 2.2651 | 2.2400 |
| parameter 2 |  | 12.7870 | 0.2022 | 0.8021 | 0.04368 | 0.7665 | 9.4840 |
| parameter 3 |  |  |  | -0.383 |  |  |  |
| AIC | 1002.4850 | 964.9006 | 955.9563 | 945.3735 | 988.7944 | 945.0411* | 947.5190 |
| BIC | 1005.8922 | 971.7149 | 962.7707 | 955.5950 | 995.6088 | 951.8555* | 954.3333 |
| **Bevacizumab and chemotherapy-OS** | | | | | | | |
| parameter 1 | 0.02707 | 1.53 | 1.9529 | 2.8963 | 0.03629 | 3.1464 | 1.8670 |
| parameter 2 |  | 30.140 | 0.0697 | 1.0101 | 0.0177 | 0.9117 | 22.9210 |
| parameter 3 |  |  |  | -0.7349 |  |  |  |
| AIC | 951.5111 | 932.2552 | 927.8778 | 918.0204* | 945.3777 | 919.0047 | 924.7727 |
| BIC | 954.9183 | 939.0696 | 934.6921 | 928.2419* | 952.1921 | 925.8191 | 931.5870 |
| *best fitted model; AIC, Akaike information criterion; BIC, Bayesian Information Criterion; PFS, progression-free survival; OS, overall survival. | | | | | | |  |

**Supplementary Table 4. Long-term PFS and OS data**

| Cycle  (3 week) | PFS | | OS | |
| --- | --- | --- | --- | --- |
|  | CBC | BC | CBC | BC |
| 0 | 100.00% | 100.00% | 100.00% | 100.00% |
| 1 | 99.67% | 99.97% | 99.85% | 100.00% |
| 2 | 98.77% | 99.44% | 99.53% | 99.99% |
| 3 | 97.36% | 97.76% | 99.08% | 99.90% |
| 4 | 95.51% | 94.85% | 98.51% | 99.56% |
| 5 | 93.28% | 90.98% | 97.84% | 98.84% |
| 6 | 90.73% | 86.47% | 97.08% | 97.72% |
| 7 | 87.93% | 81.61% | 96.24% | 96.26% |
| 8 | 84.95% | 76.62% | 95.34% | 94.53% |
| 9 | 81.83% | 71.66% | 94.37% | 92.60% |
| 10 | 78.63% | 66.83% | 93.34% | 90.55% |
| 11 | 75.41% | 62.21% | 92.27% | 88.41% |
| 12 | 72.19% | 57.83% | 91.15% | 86.24% |
| 13 | 69.01% | 53.71% | 90.00% | 84.05% |
| 14 | 65.89% | 49.85% | 88.81% | 81.89% |
| 15 | 62.87% | 46.27% | 87.60% | 79.76% |
| 16 | 59.94% | 42.94% | 86.37% | 77.67% |
| 17 | 57.12% | 39.86% | 85.12% | 75.64% |
| 18 | 54.42% | 37.01% | 83.85% | 73.66% |
| 19 | 51.84% | 34.38% | 82.57% | 71.75% |
| 20 | 49.39% | 31.95% | 81.29% | 69.90% |
| 21 | 47.05% | 29.71% | 80.00% | 68.12% |
| 22 | 44.84% | 27.65% | 78.71% | 66.40% |
| 23 | 42.75% | 25.74% | 77.43% | 64.74% |
| 24 | 40.76% | 23.98% | 76.15% | 63.14% |
| 25 | 38.89% | 22.36% | 74.87% | 61.60% |
| 26 | 37.12% | 20.86% | 73.60% | 60.12% |
| 27 | 35.45% | 19.48% | 72.34% | 58.70% |
| 28 | 33.87% | 18.19% | 71.10% | 57.32% |
| 29 | 32.38% | 17.01% | 69.86% | 56.00% |
| 30 | 30.98% | 15.91% | 68.64% | 54.73% |
| 31 | 29.65% | 14.90% | 67.44% | 53.50% |
| 32 | 28.40% | 13.96% | 66.25% | 52.32% |
| 33 | 27.22% | 13.09% | 65.08% | 51.19% |
| 34 | 26.10% | 12.28% | 63.92% | 50.09% |
| 35 | 25.04% | 11.52% | 62.78% | 49.03% |
| 36 | 24.04% | 10.83% | 61.66% | 48.01% |
| 37 | 23.10% | 10.18% | 60.56% | 47.03% |
| 38 | 22.20% | 9.57% | 59.48% | 46.07% |
| 39 | 21.36% | 9.01% | 58.42% | 45.16% |
| 40 | 20.55% | 8.48% | 57.38% | 44.27% |
| 41 | 19.79% | 7.99% | 56.35% | 43.41% |
| 42 | 19.07% | 7.54% | 55.35% | 42.58% |
| 43 | 18.38% | 7.11% | 54.36% | 41.78% |
| 44 | 17.73% | 6.71% | 53.40% | 41.00% |
| 45 | 17.11% | 6.34% | 52.45% | 40.25% |
| 46 | 16.52% | 5.99% | 51.52% | 39.52% |
| 47 | 15.96% | 5.66% | 50.61% | 38.81% |
| 48 | 15.43% | 5.36% | 49.72% | 38.13% |
| 49 | 14.92% | 5.07% | 48.85% | 37.46% |
| 50 | 14.44% | 4.80% | 48.00% | 36.82% |
| 51 | 13.98% | 4.55% | 47.16% | 36.20% |
| 52 | 13.53% | 4.31% | 46.35% | 35.59% |
| 53 | 13.11% | 4.09% | 45.55% | 35.00% |
| 54 | 12.71% | 3.88% | 44.77% | 34.43% |
| 55 | 12.33% | 3.68% | 44.00% | 33.87% |
| 56 | 11.96% | 3.50% | 43.25% | 33.33% |
| 57 | 11.61% | 3.32% | 42.52% | 32.81% |
| 58 | 11.27% | 3.16% | 41.80% | 32.30% |
| 59 | 10.94% | 3.00% | 41.10% | 31.80% |
| 60 | 10.63% | 2.86% | 40.41% | 31.32% |
| 61 | 10.34% | 2.72% | 39.74% | 30.84% |
| 62 | 10.05% | 2.59% | 39.09% | 30.39% |
| 63 | 9.78% | 2.47% | 38.44% | 29.94% |
| 64 | 9.52% | 2.35% | 37.82% | 29.50% |
| 65 | 9.26% | 2.24% | 37.20% | 29.08% |
| 66 | 9.02% | 2.14% | 36.60% | 28.67% |
| 67 | 8.79% | 2.04% | 36.01% | 28.26% |
| 68 | 8.56% | 1.94% | 35.44% | 27.87% |
| 69 | 8.35% | 1.86% | 34.87% | 27.49% |
| 70 | 8.14% | 1.77% | 34.32% | 27.11% |
| 71 | 7.94% | 1.69% | 33.79% | 26.74% |
| 72 | 7.74% | 1.62% | 33.26% | 26.39% |
| 73 | 7.56% | 1.55% | 32.74% | 26.04% |
| 74 | 7.38% | 1.48% | 32.24% | 25.70% |
| 75 | 7.20% | 1.41% | 31.75% | 25.37% |
| 76 | 7.04% | 1.35% | 31.26% | 25.04% |
| 77 | 6.87% | 1.29% | 30.79% | 24.72% |
| 78 | 6.72% | 1.24% | 30.33% | 24.41% |
| 79 | 6.57% | 1.19% | 29.87% | 24.11% |
| 80 | 6.42% | 1.14% | 29.43% | 23.81% |
| 81 | 6.28% | 1.09% | 29.00% | 23.52% |
| 82 | 6.14% | 1.04% | 28.57% | 23.23% |
| 83 | 6.01% | 1.00% | 28.15% | 22.95% |
| 84 | 5.88% | 0.96% | 27.75% | 22.68% |
| 85 | 5.76% | 0.92% | 27.35% | 22.41% |
| 86 | 5.64% | 0.88% | 26.96% | 22.15% |
| 87 | 5.52% | 0.85% | 26.57% | 21.90% |
| 88 | 5.41% | 0.82% | 26.20% | 21.64% |
| 89 | 5.30% | 0.78% | 25.83% | 21.40% |
| 90 | 5.19% | 0.75% | 25.47% | 21.16% |
| 91 | 5.09% | 0.72% | 25.12% | 20.92% |
| 92 | 4.99% | 0.69% | 24.77% | 20.69% |
| 93 | 4.89% | 0.67% | 24.43% | 20.46% |
| 94 | 4.80% | 0.64% | 24.10% | 20.24% |
| 95 | 4.70% | 0.62% | 23.77% | 20.02% |
| 96 | 4.61% | 0.59% | 23.45% | 19.81% |
| 97 | 4.53% | 0.57% | 23.14% | 19.60% |
| 98 | 4.44% | 0.55% | 22.83% | 19.39% |
| 99 | 4.36% | 0.53% | 22.53% | 19.19% |
| 100 | 4.28% | 0.51% | 22.24% | 18.99% |
| 101 | 4.20% | 0.49% | 21.95% | 18.79% |
| 102 | 4.13% | 0.47% | 21.66% | 18.60% |
| 103 | 4.06% | 0.46% | 21.39% | 18.41% |
| 104 | 3.98% | 0.44% | 21.11% | 18.23% |
| 105 | 3.92% | 0.42% | 20.84% | 18.04% |
| 106 | 3.85% | 0.41% | 20.58% | 17.87% |
| 107 | 3.78% | 0.39% | 20.32% | 17.69% |
| 108 | 3.72% | 0.38% | 20.07% | 17.52% |
| 109 | 3.65% | 0.37% | 19.82% | 17.35% |
| 110 | 3.59% | 0.35% | 19.58% | 17.18% |
| 111 | 3.53% | 0.34% | 19.34% | 17.02% |
| 112 | 3.48% | 0.33% | 19.10% | 16.86% |
| 113 | 3.42% | 0.32% | 18.87% | 16.70% |
| 114 | 3.36% | 0.31% | 18.64% | 16.54% |
| 115 | 3.31% | 0.30% | 18.42% | 16.39% |
| 116 | 3.26% | 0.29% | 18.20% | 16.24% |
| 117 | 3.21% | 0.28% | 17.99% | 16.09% |
| 118 | 3.16% | 0.27% | 17.78% | 15.94% |
| 119 | 3.11% | 0.26% | 17.57% | 15.80% |
| 120 | 3.06% | 0.25% | 17.36% | 15.66% |
| 121 | 3.01% | 0.24% | 17.16% | 15.52% |
| 122 | 2.97% | 0.23% | 16.97% | 15.38% |
| 123 | 2.92% | 0.23% | 16.77% | 15.25% |
| 124 | 2.88% | 0.22% | 16.58% | 15.12% |
| 125 | 2.84% | 0.21% | 16.40% | 14.99% |
| 126 | 2.79% | 0.21% | 16.21% | 14.86% |
| 127 | 2.75% | 0.20% | 16.03% | 14.73% |
| 128 | 2.71% | 0.19% | 15.85% | 14.61% |
| 129 | 2.67% | 0.19% | 15.68% | 14.48% |
| 130 | 2.64% | 0.18% | 15.51% | 14.36% |
| 131 | 2.60% | 0.17% | 15.34% | 14.24% |
| 132 | 2.56% | 0.17% | 15.17% | 14.12% |
| 133 | 2.53% | 0.16% | 15.01% | 14.01% |
| 134 | 2.49% | 0.16% | 14.85% | 13.89% |
| 135 | 2.46% | 0.15% | 14.69% | 13.78% |
| 136 | 2.42% | 0.15% | 14.53% | 13.67% |
| 137 | 2.39% | 0.14% | 14.38% | 13.56% |
| 138 | 2.36% | 0.14% | 14.23% | 13.45% |
| 139 | 2.33% | 0.14% | 14.08% | 13.35% |
| 140 | 2.29% | 0.13% | 13.94% | 13.24% |
| 141 | 2.26% | 0.13% | 13.79% | 13.14% |
| 142 | 2.23% | 0.12% | 13.65% | 13.04% |
| 143 | 2.21% | 0.12% | 13.51% | 12.94% |
| 144 | 2.18% | 0.12% | 13.37% | 12.84% |
| 145 | 2.15% | 0.11% | 13.24% | 12.74% |
| 146 | 2.12% | 0.11% | 13.11% | 12.64% |
| 147 | 2.09% | 0.11% | 12.98% | 12.55% |
| 148 | 2.07% | 0.10% | 12.85% | 12.45% |
| 149 | 2.04% | 0.10% | 12.72% | 12.36% |
| 150 | 2.02% | 0.10% | 12.59% | 12.27% |
| 151 | 1.99% | 0.09% | 12.47% | 12.18% |
| 152 | 1.97% | 0.09% | 12.35% | 12.09% |
| 153 | 1.94% | 0.09% | 12.23% | 12.00% |
| 154 | 1.92% | 0.09% | 12.11% | 11.91% |
| 155 | 1.90% | 0.08% | 12.00% | 11.83% |
| 156 | 1.87% | 0.08% | 11.88% | 11.74% |
| 157 | 1.85% | 0.08% | 11.77% | 11.66% |
| 158 | 1.83% | 0.08% | 11.66% | 11.58% |
| 159 | 1.81% | 0.08% | 11.55% | 11.49% |
| 160 | 1.79% | 0.07% | 11.44% | 11.41% |
| 161 | 1.77% | 0.07% | 11.34% | 11.33% |
| 162 | 1.74% | 0.07% | 11.23% | 11.25% |
| 163 | 1.72% | 0.07% | 11.13% | 11.18% |
| 164 | 1.71% | 0.07% | 11.03% | 11.10% |
| 165 | 1.69% | 0.06% | 10.93% | 11.02% |
| 166 | 1.67% | 0.06% | 10.83% | 10.95% |
| 167 | 1.65% | 0.06% | 10.73% | 10.88% |
| 168 | 1.63% | 0.06% | 10.63% | 10.80% |
| 169 | 1.61% | 0.06% | 10.54% | 10.73% |
| 170 | 1.59% | 0.06% | 10.44% | 10.66% |
| 171 | 1.58% | 0.05% | 10.35% | 10.59% |
| 172 | 1.56% | 0.05% | 10.26% | 10.52% |
| 173 | 1.54% | 0.05% | 10.17% | 10.45% |
| CBC, cadonilimab plus bevacizumab and chemotherapy; BC, bevacizumab and chemotherapy; PFS, progression-free survival; OS, overall survival. | | | | |

**Supplementary Table 5.** Scenario analysis results

| Strategy | Total costs, $ | Total QALYs | Incremental Costs, $ | Incremental Qalys | ICER |
| --- | --- | --- | --- | --- | --- |
| **Discount of cadonilimab：72%** | | | | | |
| BC group | 44,108.30 | 2.22 | - | - | - |
| CBC group | 57,624.54 | 2.58 | 13,516.24 | 0.36 | 37,803.74 |
| **At the 5-year time horizon** | | | | | |
| BC group | 39,742.82 | 1.82 | - | - | - |
| CBC group | 71,443.40 | 2.13 | 31,700.57 | 0.31 | 101,638.04 |
| CBC, cadonilimab plus bevacizumab and chemotherapy; BC, bevacizumab and chemotherapy; QALYs, quality-adjusted life years; ICER, incremental cost-effectiveness ratio. | | | | | |


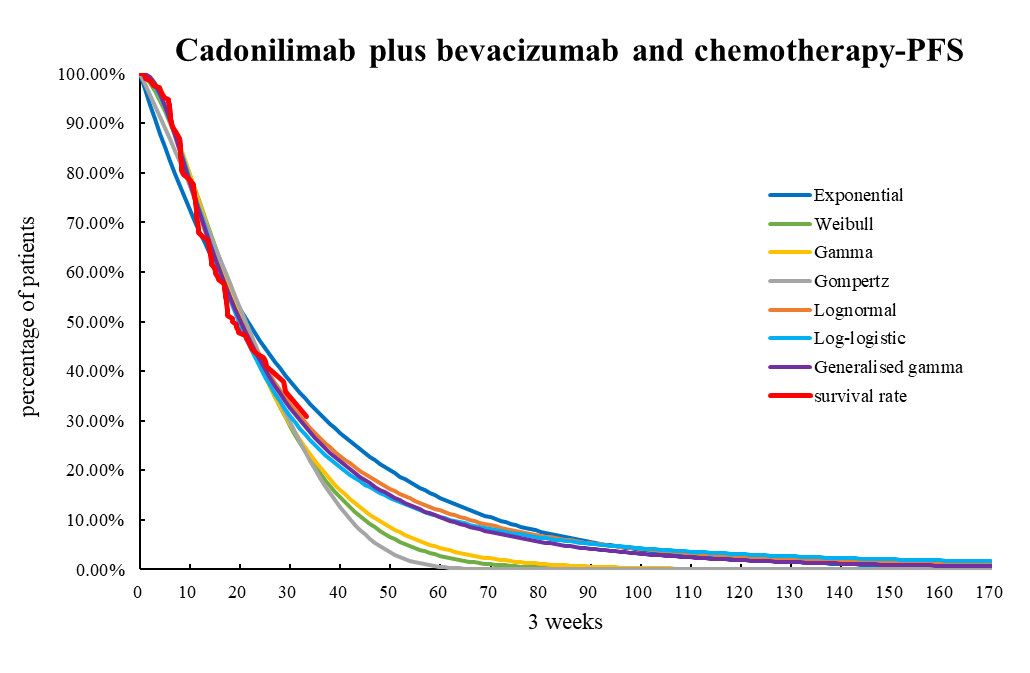


**Supplementary Figure 1.** The reconstructed Kaplan-Meier PFS curves of cadonilimab plus bevacizumab and chemotherapy


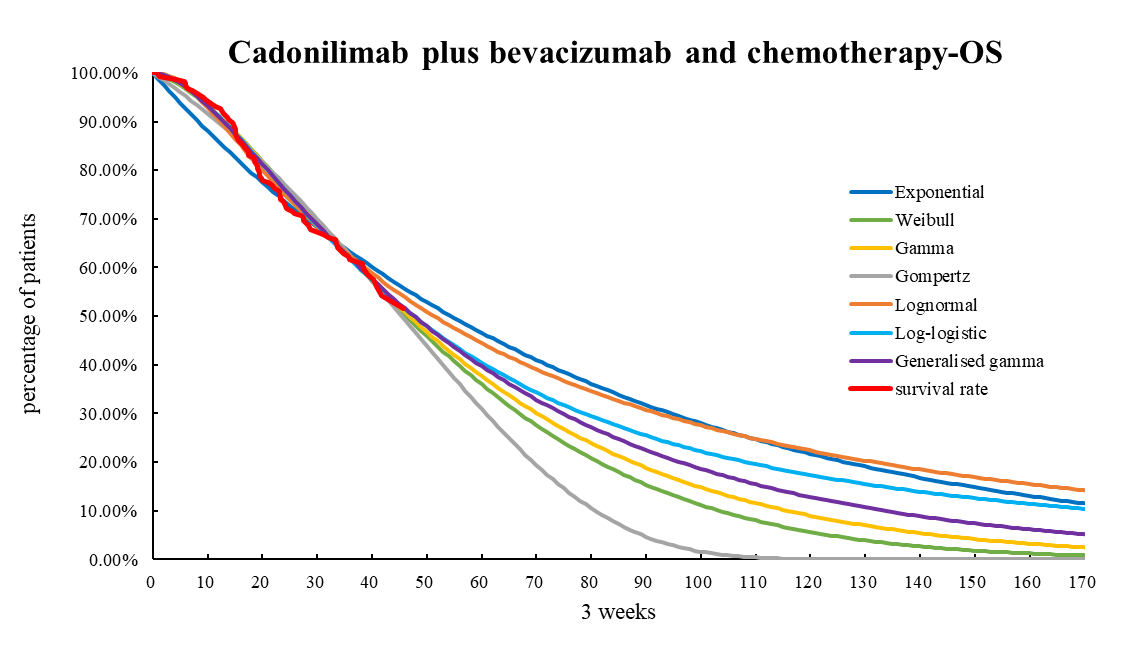


**Supplementary Figure 2.** The reconstructed Kaplan-Meier OS curves of cadonilimab plus bevacizumab and chemotherapy


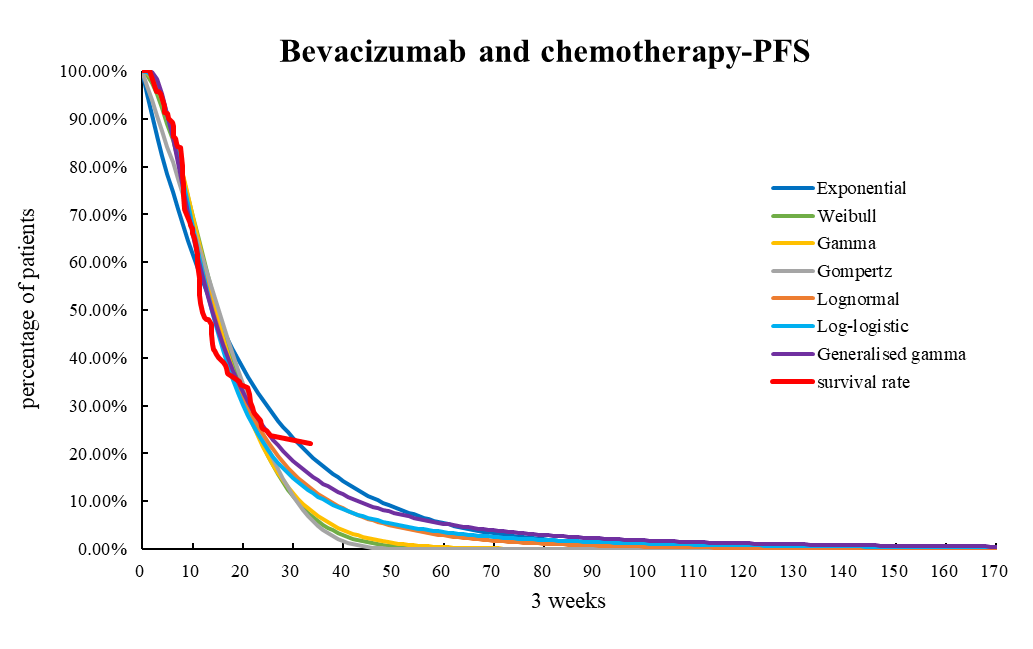
**Supplementary Figure 3.** The reconstructed Kaplan-Meier PFS curves of bevacizumab and chemotherapy


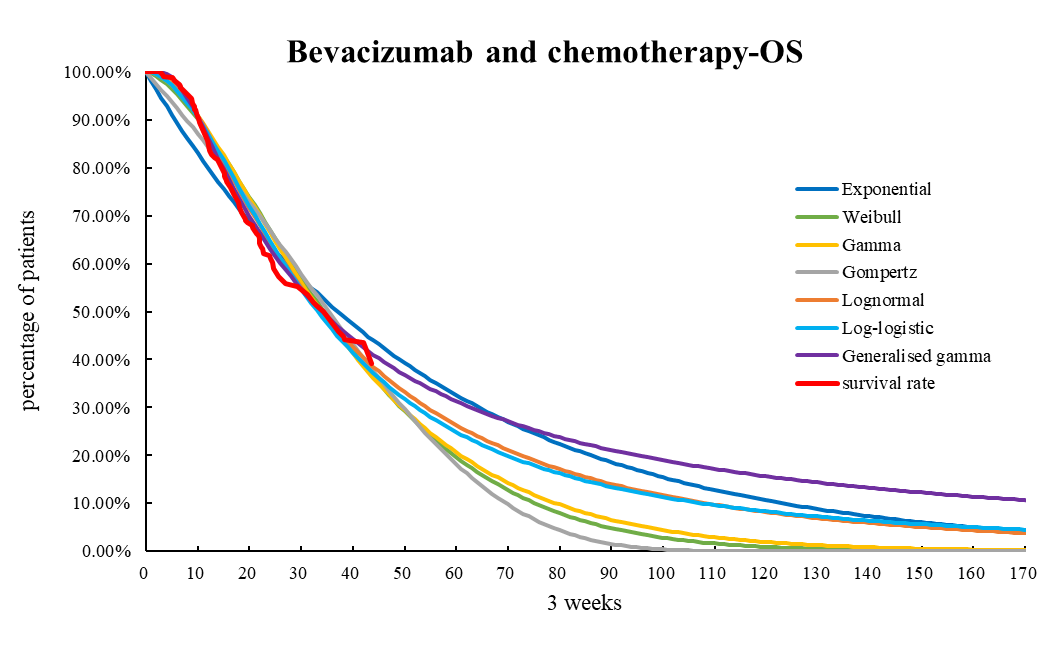
**Supplementary Figure 4.** The reconstructed Kaplan-Meier OS curves of bevacizumab and chemotherapy
